# Editorial: Transcriptional and post-transcriptional regulations in agricultural species after stresses

**DOI:** 10.3389/fgene.2022.1127832

**Published:** 2023-01-04

**Authors:** Suxu Tan, Rani Alex, Turgay Unver

**Affiliations:** ^1^ Institute of Aquatic Biotechnology, College of Life Sciences, Qingdao University, Qingdao, China; ^2^ ICAR-National Dairy Research Institute, Karnal, Haryana, India; ^3^ Ficus Biotechnology, Ankara, Türkiye; ^4^ Faculty of Engineering, Ostim Technical University, Ankara, Türkiye

**Keywords:** agriculture, transcriptiome, post-transcriptional regulation, stress, gene

According to the announcement of United Nations (https://www.un.org), the world population hit 8 billion on 15 November 2022, which could reach a whopping 10 billion people by 2050. Agriculture is the very basic foundation of the modern human society and civilization. In a broad sense, agriculture comprises planting, animal husbandry, aquaculture, and forestry. Agricultural species provide food, feed, fiber, fuel, etc., on which we rely to nourish and sustain ourselves and the next-generation. Agricultural species, however, face various stresses during development, including but not limited to bacteria, viruses, drought, temperature, feed, and chemicals. Each of these adverse conditions may compromise physiological and cellular functions, as well as activate the reprogramming of transcription to maintain the homeostasis of an individual. Understanding the molecular mechanisms and regulations underlying these processes could help select breeding populations, genetically modify organisms, facilitate diagnosis, and improve welfare, ultimately benefiting human society and the whole world.

Referring to the central dogma of molecular biology (Crick, 1970), phenotype can be determined and regulated at multiple layers including the DNA, RNA, and protein level. In response to environmental cues and changing conditions, the RNA-level transcriptional and post-transcriptional regulations come into effect swiftly as pioneers. This Research Topic focuses on the transcriptional and post-transcriptional regulations in agricultural species responding/adapting to various stresses, aiming to reveal the underlying molecular mechanisms and provide insights into their applications in practice.

This Research Topic covers a wide spectrum of agricultural species, including livestock (cattle, sheep, pig, and chicken), fish (Atlantic salmon), cereals and legumes (wheat, maize, soybean, and pea), vegetable (eggplant), fruit (pear), and ornamental plants (red plum, lily, and lotus), in response to various stresses and conditions, such as heat, cold, drought, hypoxia, bacteria, chemicals, weaning, and mastitis. Most studies in this Research Topic examined the whole transcriptome to reveal the underlying molecular mechanisms following stresses. For example, in the context of global warming, Niu et al. employed transcriptome sequencing to study the mechanism by which high temperature affect grain abortion of maize, by comparing heat-resistant and heat-sensitive variety under a 7-day heat stress treatment after pollination. They unveiled that heat stress mainly resulted in reduced carbohydrate availability for grain development, leading to reduced kernel number.

In addition to the transcriptome profiling, Nayan et al. utilized MeDIP-Seq to investigate the genome-wide methylation specific to pathogen-caused subclinical mastitis in water buffalo and its consequential effect on the gene expression landscape. Furthermore, the authors built a Buffalo Subclinical Mastitis Methylome-Transcriptome database (BSCM2TDb) to catalogue the results of this study, which could help buffalo breeders in breed improvement and disease management programs.

Some articles focus on the studies of gene families, instead of the whole transcriptome. For instance, Wang et al. identified 719 putative Type 2C protein phosphatase (PP2C) genes in eight Rosaceae species (Chinese white pear, European pear, Japanese apricot, apple, peach, strawberry, sweet cherry, and black raspberry), and further studied the evolution of PP2C genes. Moreover, the authors performed gene expression analyses specifically in Chinese white pear and identified candidate genes that participated in stress responses, including heat, cold, drought, NaCl, and abscisic acid.

Researchers of this Research Topic also studied post-transcriptional regulations, such as the profiles of lncRNA, miRNA, and circRNA in Tibetan sheep, hybrid hens, and Duroc pigs, respectively. For example, Lu et al. revealed the regulatory mechanisms of the lncRNAs and mRNAs in the adaptation of Tibetan sheep to hypoxia, by comparing lung tissues from high-altitude Tibetan sheep and low-altitude Hu sheep. Functional enrichment and interaction network analysis results showed that lncRNA and mRNA may adapt to hypoxia *via* lipid metabolism, facilitating further analyses of plateau adaptability.

This Research Topic produced vast and valuable data and information, identified crucial biological pathways and candidate genes, and increased our knowledge of the underlying mechanisms of stress responses in agricultural species. These laid a solid foundation to move forward to use genomic breeding approaches, including marker-assisted selection, genomic selection and editing, to speed breeding of resistant populations and subspecies. In addition, the enriched transcriptomics data of this Research Topic, integrated with other omics data of genomics, proteomics, metabolomics, and phenomics, i.e., multi-omics strategies with systems biology, could enhance our understanding and accelerate the breeding improvement (Yang et al., 2021). Furthermore, organic and precision agricultural systems coupled with artificial intelligence have the potential to achieve a balance between productivity, nutrition, economic development, social welfare, and environmental impact (Gebbers and Adamchuk, 2010; Reganold and Wachter, 2016).

We would like to thank all authors and reviewers who contribute to this Research Topic. Besides, we want to pay tribute to agricultural practitioners for their hard work, especially the sacrifices of our father and mother, grandfather and grandmother, extending to our ancestors, who tried their best to feed us well and make our lives better, with the help of agriculture. Similarly, we need to do more to ensure our children have enough food in the future and make them proud of us. To achieve the daunting task, concerted efforts of farmers, researchers, engineers, and policymakers are much-needed.

## References

[B1] Crick F. (1970). Central dogma of molecular biology. Nature 227, 561–563. 10.1038/227561a0 4913914

[B2] Gebbers R. Adamchuk V. I. (2010). Precision agriculture and food security. Science 327, 828–831. 10.1126/science.1183899 20150492

[B3] Reganold J. P. Wachter J. M. (2016). Organic agriculture in the twenty-first century. Nat. Plants 2, 15221. 10.1038/nplants.2015.221 27249193

[B4] Yang Y. Saand M. A. Huang L. Abdelaal W. B. Zhang J. Wu Y. (2021). Applications of multi-omics technologies for crop improvement. Front. Plant Sci. 12, 563953. 10.3389/fpls.2021.563953 34539683 PMC8446515

